# A Case of Transient Hypercalcemia Following Fistulization of a Calcified Mitral Annulus

**DOI:** 10.1210/jcemcr/luad169

**Published:** 2024-01-04

**Authors:** Kobi Jacob Perl, Michal Julius, Dror Cantrell

**Affiliations:** Department “C” of Internal Medicine, Shamir Medical Center, Sackler Medical School Tel Aviv University, Tzrifin 70300, Israel; Department “C” of Internal Medicine, Shamir Medical Center, Sackler Medical School Tel Aviv University, Tzrifin 70300, Israel; Department “C” of Internal Medicine, Shamir Medical Center, Sackler Medical School Tel Aviv University, Tzrifin 70300, Israel

**Keywords:** mitral annulus calcification (MAC), caseous mitral annular calcification (CMAC), liquefaction necrosis, hypercalcemia, hyperphosphatemia

## Abstract

We report a case of severe symptomatic hypercalcemia that resolved after a short course of therapy of exclusively fluids and furosemide. An extensive workup for metabolic, neoplastic, and drug-induced causes did not provide a possible etiology of the hypercalcemia. After calcium level returned to baseline, the patient was discharged, only to return a week later with multiple embolic strokes of unknown source. The comparison of cardiac imaging obtained during the hospitalization periods established a possible mechanism for both phenomena; the interior caseous cavity of a calcified mitral annulus (CMAC), which was demonstrated on echocardiography during the first hospitalization, disappeared in a subsequent study in the second hospitalization, probably reflecting a fistulization of the structure into the left ventricle. The spill of contents into the bloodstream, over several days presumably, explains the transient increase in calcium, and the embolic events that followed. We hereby demonstrate a clear relationship between the fistulization of a CMAC and hypercalcemia, emphasizing the risks of this valvular pathology, and introducing a rare mechanism for transient and potentially severe hypercalcemia.

## Introduction

Hypercalcemia is a common medical problem with numerous etiologies. More than 90% of cases are caused by primary hyperparathyroidism or malignancy. A mini review by Motlaghzadeh et al describes rare causes of hypercalcemia [1]. Although uncommon, most originate through known mediators, including 1,25-dihydroxyvitamin D, parathyroid hormone (PTH), and PTH-related protein. One remarkable mechanism, which plays a part in the transient hypercalcemia observed in patients with acute renal failure secondary to rhabdomyolysis, does not involve any of these mediators, but rather the remobilization of calcium from damaged tissue. This mechanism demonstrates the possibility of transient hypercalcemia caused by a shift between compartments.

Mitral annular calcification (MAC) is a degenerative chronic process causing calcium deposits at the base of the mitral valve [2]. It is a relatively common finding, reported in as many as 8% to 15% of adults. Caseous calcification of the mitral annulus (CMAC) is a rare variant of MAC, in which the inner content of the perivalvular mass undergoes liquefaction necrosis [3].

The echocardiographic prevalence of CMAC is approximately 0.6% in patients with MAC and is higher in older women, hypertensive patients, and patients with chronic kidney disease or altered calcium-phosphate metabolism. On echocardiography, this lesion can be identified as an echo-dense mass with a central lucent area, typically located in the posterior annular region [3]. On gross pathology, it appears as a perivalvular collection of amorphous, white material with a “toothpaste-like” texture. The main components of this material are calcium, salts, fatty acids, and cholesterol.

CMAC is often an incidental finding and was originally described as benign. However, it may cause different sorts of valvular dysfunction, and a few reports also linked it with embolic events, including cerebrovascular accidents, retinal artery occlusion, and acute coronary syndrome [4]. Suggested mechanisms for embolization include thrombus formation, dehiscence of small calcified parts, or fistulization of the caseous cavity in the lumen of the left ventricle [3]. The latter two involve the release of calcium from the valvular calcification into the bloodstream, and thus can, in theory, cause a short-term elevation of blood calcium levels.

Here we describe a case of transient symptomatic hypercalcemia and multiple embolic events that corresponded to liquefaction necrosis and fistulization of a CMAC. We believe that it is the disintegration of the structure that mobilized calcium into the blood and led to the dangerous electrolyte imbalance. Cardiologists and endocrinologists both should be informed of this near-fatal and unfamiliar complication of CMAC.

## Case Presentation

A 68-year-old woman presented to the emergency department with acute-onset confusion and stupor. Several days before she complained of constipation, and the night prior to admission she reported abdominal pain, vomiting, and diarrhea. Her past medical history included hypothyroidism, hypertension, osteoporosis, and diabetes.

## Diagnostic Assessment

On admission, the patient’s vital signs were within normal limits. Somnolence was the only remarkable neurological finding. Initial laboratory tests were notable for a high serum calcium of 15.1 mg/dL (3.77 mmol/L) (normal, 8.6-10.3 mg/dL [2.15-2.57 mmol/L]). Once other causes of reduced consciousness were ruled out, through noncontrast head computed tomography (CT), lumbar puncture, and urine toxicology screen, her situation was attributed to the hypercalcemic state.

During hospitalization, further workup for hypercalcemia revealed marked hyperphosphatemia of 7.0 mg/dL (2.3 nmol/L) (normal, 2.8-4.5 mg/dL [0.9-1.5 nmol/L]), suppressed PTH level of 0.9 pmol/L (normal, 1.6-6.9 pmol/L), and a low vitamin D-25(OH) level of 15 ng/mL (37.5 nmol/L) (normal, 20-50 ng/mL [50-125 nmol/L]). Notably, creatinine levels were within the normal range 0.95 mg/dL (84 μmol/L) (normal, 0.6-1.1 mg/dL [53-97 μmol/L]). A review of diet and medications did not reveal a causative agent, and serum protein electrophoresis was negative for a monoclonal protein spike.

**Figure 1.**
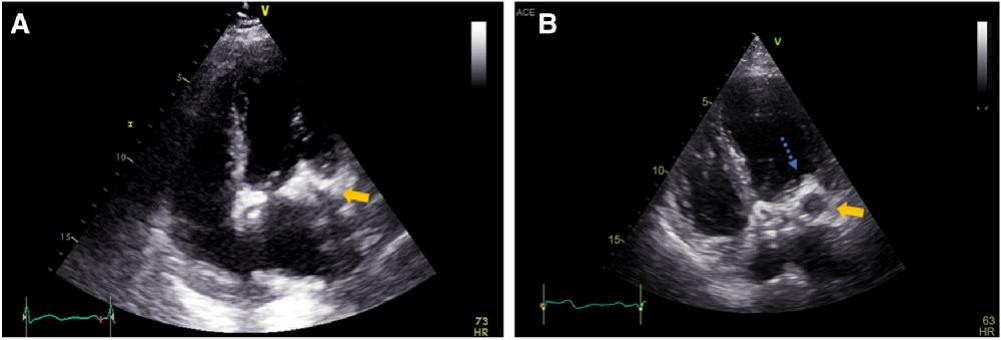
Transthoracic ultrasound, 4-chamber view, during the first and second hospitalizations (A and B, respectively). A hyperechoic cardiac mass on the mitral annulus is demonstrated in both studies (yellow solid arrows), with a clearing in the interior part of the mass, and an appearance of small mobile vegetation (blue dotted arrow), in the latter.

Another laboratory finding was an elevated cardiac troponin-T level of 0.34 μg/L (normal, 0-0.013 μg/L). This sign of myocardial injury was not accompanied by complaints of chest pain or an ischemic pattern on the electrocardiogram. A full-body CT scan did not demonstrate any findings suggesting malignancy. However, a calcified amorphous tumor within the left ventricle, measuring 4.6 cm in diameter, was noted. This finding was further characterized by echocardiography, which demonstrated a caseous formation involving the posterior commissure of the mitral valve (Fig. 1A). Interestingly, this structure was hemodynamically meaningful, as it caused an elevated diastolic pressure gradient. A cardiac magnetic resonance imaging (MRI) scan confirmed the presence of CMAC, and in addition, demonstrated scarring and atrophy of the adjacent myocardium.

## Treatment

While in the emergency department, the patient was treated with normal saline and antiemetics. On admission, treatment continued with the administration of intravenous fluids, and subsequently furosemide.

## Outcome and Follow-up

Repeated laboratory work 2 days after the initial presentation revealed normalization of calcium and phosphorous levels to 9.6 and 2.2 mg/dL, respectively (2.4 and 0.71 nmol/L, respectively). The patient gradually recovered to her baseline mental state. Once aware of her situation, she reported new-onset neurological symptoms including blurry vision and dizziness. The complaints were attributed to hypertensive retinopathy found on a fundoscopic examination. The patient was then discharged and referred to a neuro-ophthalmologist for further evaluation.

After discharge, the patient continued to experience worsening visual symptoms. At an ambulatory follow-up less than a week post discharge, the patient was reported to have rotatory nystagmus and was subsequently readmitted for further workup. Laboratory examinations obtained during this admission were all within normal limits, including calcium and phosphorus levels. A brain MRI displayed findings consistent with multiple thromboembolic events. Some lesions located in the cerebellum provide a typical source for central nystagmus.

A transesophageal echocardiogram revealed a clearing of the internal part of the mass and a reduction in the calcium content compared to imaging obtained during her previous admission (Fig. 1B). A new mobile vegetation of up to 0.5 mm in diameter on the ventricular aspect of the calcification was also observed. We concluded the CMAC to be the embolic source, as an infection was ruled out, and no alternative source of emboli was identified.

A cardiothoracic surgical consult concluded that the finding did not necessitate a surgical excision, as it posed little threat of recurrent embolization. Over several days of observation, the patient's symptoms improved. She was discharged with indefinite therapy of warfarin. She continued follow-up for over 2 years with no return of neurological symptoms or electrolyte abnormalities. In a newer echocardiogram, 2 years following her initial admission, the caseous calcification of the mitral annulus was still present, without the small mobile vegetation.

## Discussion

Our patient presented with severe, symptomatic hypercalcemia, as well as marked hyperphosphatemia. Both electrolyte abnormalities disappeared within several days of standard therapy. An extensive workup for metabolic, drug-induced, and neoplastic causes did not provide an explanation for the hypercalcemia. More specifically, the patient did not use any form of vitamin D supplements, diuretics, or lithium, nor did she ingest large amounts of absorbable alkali. PTH levels were suppressed, thereby excluding hyperparathyroidism. Similarly, low vitamin D-25(OH) excluded hypervitaminosis D. Other mediators of hypercalcemia, such as PTH-related protein and vitamin D-1,25(OH), were not measured. Nonetheless, we made an effort to rule out other malignancies and granulomatous diseases by performing a full-body CT scan. We also tested the serum for monoclonal protein in search of multiple myeloma. While none of these tests can fully rule out malignancies or granulomatous diseases, a follow up of the patient for more than 2 years did not reveal any hidden disease or additional episodes of hypercalcemia. If the patient had such a disease, the calcium levels would not likely remain normal over follow-up without any preservative treatment.

Various cardiac imaging modalities were notable for a finding consistent with CMAC. Repeated cardiac imaging at a subsequent hospitalization revealed the disappearance of the calcified interior within an interval of several days. The transient, unexplained nature of the patient's electrolyte abnormalities, taken with the abrupt reduction in the calcification, prompts us to suggest a connection between the valvular pathology and her severe metabolite abnormalities.

A thorough literature search discovered a similar case report, in which the caseous transformation of a known MAC was temporally associated with the appearance of hypercalcemia [5]. The described patient suffered likewise from a short-lived episode of severe, symptomatic hypercalcemia that, once treated, passed with no sequelae. Nevertheless, the authors of the report refrained from concluding a direct relation between the two processes.

Our case provides a stronger base of evidence for a connection between CMAC fistulization and hypercalcemia. Thanks to the advanced imaging that was performed at multiple time points relative to the appearance of the hypercalcemic state, we can build a coherent timeline that connects the two, through the release of calcium and phosphate into the bloodstream. In addition, the embolic events that followed shortly after the hypercalcemia correspond well with the known systemic embolization associated with CMAC and connect the differing clinical presentations within a short time frame. Overall, the different components of the patient's complex presentation are unlikely to be independent, suggesting a common etiology with a strong temporal connection between fistulization, hypercalcemia, and the appearance of embolic events.

The patient's initial presentation was also notable for elevated troponin in the absence of chest pain or angina equivalents. A cardiac MRI showed some damage to the myocardium adjacent to the CMAC and did not support an infarction in a specific coronary artery territory. This finding suggests that the presence of the CMAC by itself can cause injury to the nearby myocardium in a process that does not require an embolic event. Another local effect of CMAC was an increase in the pressure gradient across the valve, as measured by echocardiography.

In conclusion, this case report adds to the accumulating evidence that CMAC is not a benign condition. It highlights the risk of systemic embolization and describes an even rarer phenomenon of severe, yet transient, hypercalcemia caused by the release of calcium from the valvular calcification into the bloodstream. As the knowledge of these risks expands, it might be of benefit to reexamine the indications for excisional surgery of CMAC. Furthermore, this case highlights the possibility of hypercalcemia and hyperphosphatemia caused by a remobilization process from tissue to the bloodstream, in contrast to the much more familiar metabolically—or hormonally—mediated causes. In practice, this calls for a search for disintegrating calcium deposits in obscure cases of hypercalcemia.

## Learning Points

- Remobilization of calcium from tissue to the bloodstream can rarely cause hypercalcemia.
- Disintegration or fistulization of a CMAC can cause severe electrolyte imbalances through a spill of contents into the bloodstream.
- The hypercalcemia and hyperphosphatemia caused by such processes are transient in nature but may be severe or even fatal.
- A CMAC can inflict local myocardial injury, potentially more so during fistulization.

## Contributors

K.P. collected data and drafted the manuscript; M.J. edited the manuscript; and D.C. contributed to editing, case management, and patient follow-up. All authors reviewed and approved the final draft.

## Data Availability

Data sharing is not applicable to this article as no data sets were generated or analyzed during the current study.

## Abbreviations

CMAC: caseous mitral annular calcification
CT: computed tomography
MAC: mitral annular calcification
MRI: magnetic resonance imaging
PTH: parathyroid hormone
